# ﻿*Dendrocalamus wazibii* (Poaceae, Bambusoideae), a new bamboo species from Yunnan, China

**DOI:** 10.3897/phytokeys.263.162328

**Published:** 2025-09-18

**Authors:** Jian-Wei Li, Mao-Sheng Sun, Hao-Feng Bao, Chao-Mao Hui, Wei-Yi Liu

**Affiliations:** 1 Institute of Bamboo and Rattan Science, Southwest Forestry University, Kunming 650224, Yunnan, China Southwest Forestry University Kunming China

**Keywords:** Cangyuan County, *
Dendrocalamus
*, new bamboo species

## Abstract

*Dendrocalamus wazibii* (Bambusoideae), a new bamboo species is described and illustrated from Cangyuan County, Yunnan, China. Based on careful comparison of morphological and generative characters, we confirmed its identity as a new member of the genus *Dendrocalamus*. The morphology of this new species is similar to *D. peculiaris* and *D. tomentosus*, but can be easily distinguished from them by having culm leaf ligule 15 mm long (vs. 5–7 mm in *D. tomentosus* and 6–10 mm in *D. peculiaris*), linear culm leaf auricle (vs. absent in *D. tomentosus* and *D. peculiaris*), anther 2–2.5 mm long (vs. 2.5–3 mm in *D. tomentosus*; vs. 3–3.5 mm in *D. peculiaris*), stamen 5 mm long (vs. 6 mm in *D. tomentosus*; vs. 10 mm in *D. peculiaris*). According to the IUCN Red List Categories and Criteria (IUCN 2024), it meets the criteria to be listed as Data Deficient (DD).

## ﻿Introduction

The genus *Dendrocalamus* is native to India and represents a classic Palaeotropical woody bamboo group (Li and Hsueh 1988; Wang and Li 2019). Comprising approximately 50 species, it is distributed across tropical and subtropical regions of Asia (Bamboo Phylogeny Group 2012), including India, China, Indonesia, Sikkim, Myanmar, Vietnam and Laos (Keng and Wang 1996; Li et al. 2006), It is recognised as the second largest genus in the subtribe Bambusinae of the subfamily Bambusoideae (Nguyen et al. 2017b). There are about 30 species of *Dendrocalamus* distributed in China, primarily found in southern and south-western regions, with Yunnan Province exhibiting the highest species diversity (Keng and Wang 1996; Li et al. 2006; Yi et al. 2008; Wang and Li 2019). In recent years, several new species of *Dendrocalamus* have continued to be described and published (Mao et al. 2009; Nguyen et al. 2010, 2012, 2013a, 2013b, 2014, 2017a, 2017b; Nguyen and Le 2012; Nguyen and Xia 2013; Chen et al. 2013; Yang et al. 2015, 2016; Wang et al. 2016; Wang and Li 2019; Shi et al. 2024).

During a bamboo resource survey conducted in Lincang City, Yunnan, China in 2022, we discovered an unknown clumping bamboo species in Cangyuan County. The species exhibits erect culms with drooping apex, multiple branches per node (one dominant branch), inconspicuous culm leaf auricles, well-developed culm leaf ligule and reflexed leaf blades, characteristics consistent with *Dendrocalamus*. However, its remarkably prominent ligule with long oral setae makes it particularly distinctive. Due to incomplete data documentation and specimen collection at the initial discovery, we conducted follow-up expeditions in September 2024 and April 2025. During investigations in 2025, we observed flowering individuals of this species, providing crucial evidence for taxonomic determination. Ultimately, based on morphological characteristics of vegetative organs and inflorescences, we confirmed it as a novel, undocumented species of *Dendrocalamus* and formally described it herein.

## ﻿Material and methods

Field observations and measurements of living specimens were conducted in their natural habitat, morphological examinations of preserved specimens were performed at the Bamboo and Rattan Research Institute, Southwest Forestry University and floral dissection was completed under a stereomicroscope (AOSV HD206).

Comparative characteristics of related species were compiled from authoritative literature (e.g. Keng and Wang (1996); Li et al. (2006); Yi et al. (2008)) and recently described *Dendrocalamus* species (Mao et al. 2009; Nguyen et al. 2010, 2012, 2013a, 2013b, 2014, 2017a, 2017b; Nguyen and Le 2012; Nguyen and Xia 2013; Chen et al. 2013; Yang et al. 2015, 2016; Wang et al. 2016; Wang and Li 2019; Shi et al. 2024).

## ﻿Results

Through comparative analysis of the morphological differences in vegetative and generative organs between the new species and existing species of *Dendrocalamus*, we found that the new species belongs to the subg. Sinocalamus, it shares closest morphological affinity with *D. tomentosus* and *D. peculiaris* and its key diagnostic characteristics are summarised in Table 1.

**Table 1.** The morphological comparison of *Dendrocalamus wazibii*, *D. tomentosus* and *D. peculiaris*.

Characters	Dendrocalamus wazibii	D. tomentosus	D. peculiaris
Culm leaf ligule height	15 mm	5–7 mm	6–10 mm
Culm leaf auricle	linear, small and inconspicuous	absent	absent
Foliage blade size	12–26 cm × 2–4 cm	25–34 cm × 2.5–4.2 cm	25–40 cm × 3–5.5(10) cm
Secondary veins	6–9 pairs	8–12 pairs	9–12 pairs
Lemma size	5–7 mm × 4–5 mm	4–5 mm × 4–6 mm	7–11 mm × 7–8 mm
Interkeel width of the palea	ca. 1 mm	ca. 1 mm	ca. 1.5 mm
Anther length	2–2.5 mm	2.5–3 mm	3–3.5 mm
Stamen length	5 mm	6 mm	10 mm

## ﻿Taxonomy

### ﻿ Dendrocalamus wazibii

Taxon classificationPlantaePoalesPoaceae

C.M.Hui & J.W.Li sp. nov.

ECFE5C02-1CA9-5269-88D8-8DED826DC98A

urn:lsid:ipni.org:names:77369329-1

Figs 1, 2, 3

#### Diagnosis.

*Dendrocalamus wazibii* is similar to *D. peculiaris* and *D. tomentosus*, but can be easily distinguished from them by having culm leaf ligule 15 mm long (vs. 5–7 mm in *D. tomentosus* and 6–10 mm in *D. peculiaris*), linear culm leaf auricles (vs. absent in *D. tomentosus* and *D. peculiaris*), lemma 5–7 mm × 4–5 mm (vs. 4–5 mm × 4–6 mm in *D. tomentosus*; vs. 7–11 mm × 7–8 mm in *D. peculiaris*), anthers 2–2.5 mm long (vs. 2.5–3 mm in *D. tomentosus*; vs. 3–3.5 mm in *D. peculiaris*) and stamens 5 mm long (vs. 6 mm in *D. tomentosus*; vs. 10 mm in *D. peculiaris*) (See Table 1 for details).

#### Type.

China • Yunnan: Lincang City, Cangyuan County, Menglai Township, Papeng Village, 23°11'54.31"N, 99°14'50.58"E, 1376 m alt., 24 September 2024, *C. M. Hui, J. W. Li, H. F. Bao & C. H. Zhang 0072409* (Holotype & Isotype: SWFC!).

#### Description.

Rhizomes pachymorph. Culms erect, apex slightly nodding, 13–20 m tall, 7–12 cm diameter; internodes 30–40 cm long, initially densely white to pale brown tomentum; branching initiated from the 2^nd^ or 3^rd^ basal node, multiple branches per node with one dominant branch; basal nodes with aerial roots; intranode ca. 1 cm tall, with a ring of white tomentum; sheath scar prominent, with a ring of brown hair below. Culm sheaths coriaceous, deciduous; sheath blade 24–30 cm × 32–40 cm, 1/2–2/3 as long as internode length, margins ciliate; auricles small, linear with oral setae, deciduous; ligule arched, 15 mm tall, margins deeply lobed with brown oral setae 0.3–0.5 cm long (longer laterally, up to 1.5 cm); blades triangular, 8 cm tall, reflexed, ca. 1/3 as long as sheath proper, adaxial base covered with dark brown bristles. Ultimate branchlets with 6–8 foliage leaves. Leaf blades oblong-lanceolate, 12–25 cm × 2–4 cm, finely pubescent, secondary veins 6–9 pairs; margins finely serrulate; petioles 3–5 mm, sparsely pubescent; sheaths 5.5–8 cm, margins ciliate; auricles absent; ligule arcuate, ca. 1 mm tall, margins finely serrulate, occasionally oral setae. Flowering branches pendulous, leafless with 2–30 clusters pseudo-spikelets at each node; inflorescence clusters 1.2–2 cm in diameter; pseudo-spikelets broadly ovate, 8–12 × 5–7 mm, dark purple, with 5–6 florets. Glumes 2, ca. 3.5 mm long, ca. 2.5 mm width, margins ciliate; lemmas ovate, 5–7 × 4–5 mm, pubescent, many-veined, apex mucronate, margins ciliate; palea oblanceolate, ca. 4 × 2.2 mm, 2-keeled, keels 1 mm apart, margins and keels long ciliate; lodicules absent; stamens 6, ca. 4 mm long, anthers yellow, 2–2.5 mm long, anthers exserted when mature, apex acute, with several white hairs; filaments free, ca. 2 mm long; pistil ca. 5 mm long, hairy; ovary ovoid; stigma 1, purple, plumose. Fruit unknown.

**Figure 1.**
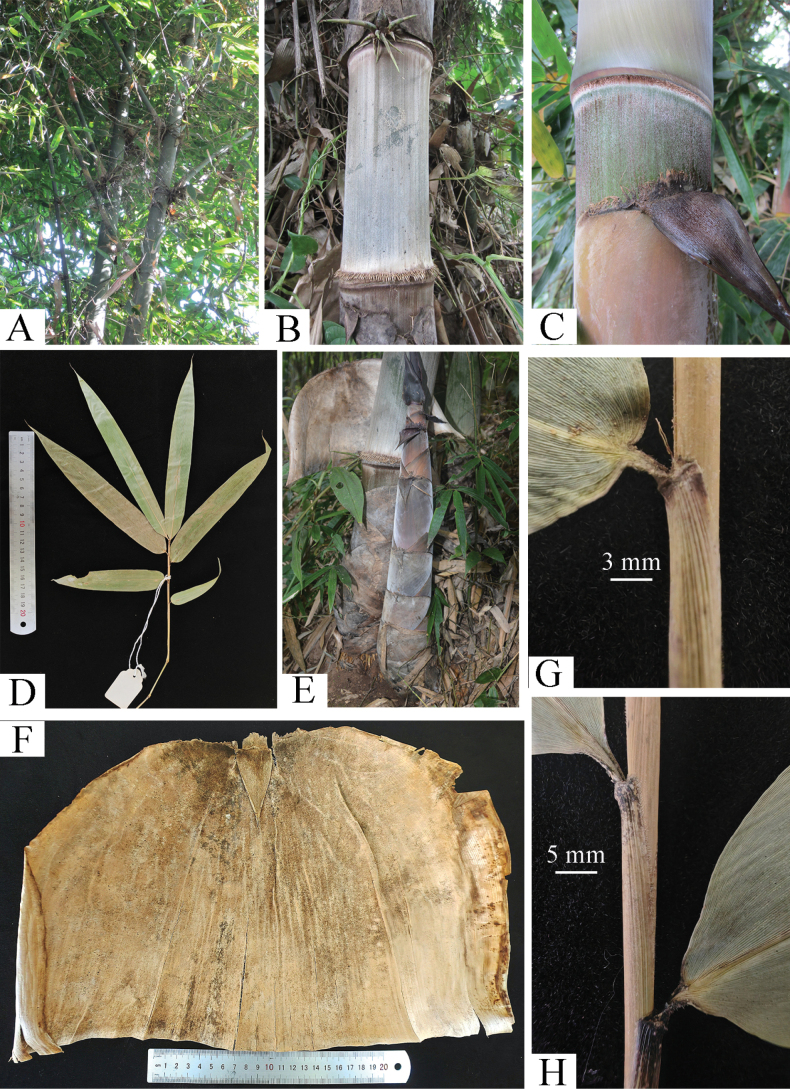
*Dendrocalamus wazibii* C.M.Hui & J.W.Li. A. Branches; B. Young culm; C. Culm leaf ligule and oral setae; D. Foliage blade; E. New shoot; F. Culm leaf sheath; G. Petiole and oral setae; H. Foliage leaf sheath.

#### Phenology.

Shooting from August to October and flowering from November to May of the next year.

#### Distribution and habitat.

*Dendrocalamus wazibii* is endemic to Yunnan, China and is currently found only near villages at an elevation of 1,376 m in Cangyuan County.

**Figure 2.**
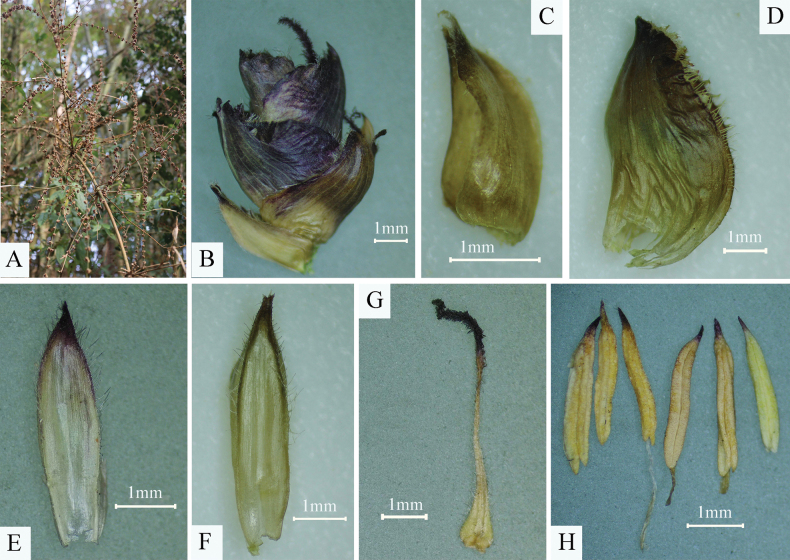
*Dendrocalamus wazibii* C.M.Hui & J.W.Li. A. Flowering branches; B. Pseudo-spikelet; C. Glume; D. Lemma; E. Lowermost palea; F. Uppermost palea; G. Pistil; H. Stamens.

#### Local uses.

The local Lahu people use its bamboo shoots for making sour bamboo shoots, while the bamboo culms are used for weaving.

#### Etymology.

The specific epithet “*wazibii*” is used in apposition to the Lahu language term “wa zi bi” that means the bamboo culms prized for their relatively high flexibility and common use in weaving.

#### Chinese name.

Huī Bái Lóng Zhú (Chinese pronunciation); 灰白龙竹 (Chinese name).

#### Conservation status.

Although we conducted three field surveys, due to the limited scope of the investigation and the presence of numerous similar habitats (e.g. vegetation type, soil, elevation etc.) near the type locality, we currently classify the new species as Data Deficient (DD), according to the IUCN (2024) criteria.

**Figure 3.**
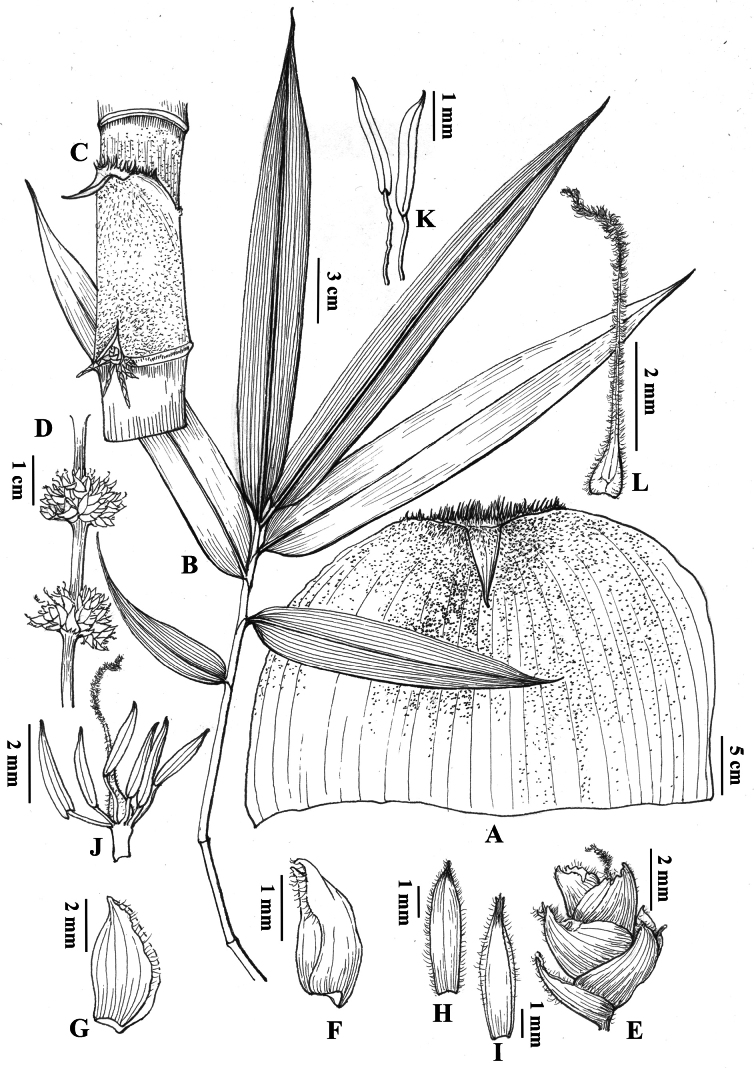
*Dendrocalamus wazibii* C.M.Hui & J.W.Li. A. Culm leaf sheath; B. Foliage blade; C. Portion of young culm with culm sheath; D. Portion of flowering branch; E. Pseudo-spikelet; F. Glume; G. Lemma; H. Lowermost palea; I. Uppermost palea; J. Pistil and stamens; K. Stamens; L. Pistil.

#### Additional specimens examined (paratypes).

China • Yunnan: Lincang City, Cangyuan County, Menglai Township, Papeng Village, 23°11'54.31"N, 99°14'50.58"E, 1376 m alt., 24 September 2024, *C. M. Hui, J. W. Li, H. F. Bao & C. H. Zhang 0072408* (SWFC!).

## ﻿Discussion

According to the classification system of Flora Reipublicae Popularis Sinicae (Keng and Wang 1996), the genus Dendrocalamus can be divided into the subg. Dendrocalamus and the subg. Sinocalamus. The diagnostic characteristics of the former are as follows: culm tip only slightly curved; mostly three dominant branches; culm leaf sheath thickly papery to thinly leathery, usually without auricles; foliage blades narrow; pseudo-spikelets numerous, clustered at the nodes of flowering branches and forming glomerules; pseudo-spikelets containing 1–5 florets; lemma apex with an aristate mucro; stigmas 1–3. The diagnostic characteristics of the latter include: culm tip drooping; one dominant branch or dominant branches indistinct; culm leaf sheaths leathery to thickly leathery; auricles small; foliage blades usually large and broad; pseudo-spikelets solitary or several borne at the nodes of flowering branches; pseudo-spikelets containing 4–8 florets; lemma apex without an aristate mucro; stigma single. The morphological characteristics of this new species are as follows: culm tips drooping; one distinct dominant branch; culm leaf sheaths leathery, caducous; auricles small; ligules reflexed; flowering branch nodes bearing clusters of 2–30 pseudo-spikelets; pseudo-spikelets containing 5–6 florets; lemma apex without an aristate mucro; stigma 1. In summary, although the characteristics of pseudo-spikelets forming glomerules in the new species conform to the typical features of subg. Dendrocalamus, its key morphological combination, such as drooping culm tips, one dominant branch, reflexed ligules, small auricles, pseudo-spikelets containing 5–6 florets and lemma without an aristate mucro, aligns more closely with the classification criteria of subg. Sinocalamus. Furthermore, based on traits including reduced auricles, reflexed ligules and the number of florets, we further assign it to sect. Sinocalamus.

Based on authoritative botanical literature (e.g. Keng and Wang (1996); Li et al. (2006); Yi et al. (2008)) and recent records of newly-described species (Mao et al. 2009; Chen et al. 2013; Yang et al. 2015, 2016; Wang et al. 2016; Wang and Li 2019; Shi et al. 2024), we have systematically revised the taxonomic groups of *Dendrocalamus* in China, confirming a total of 37 species, two varieties, five forms and one cultivar. All accepted names adhere to the standards established in Flora of China (Li et al. 2006). Using morphological evidence, we have compiled a taxonomic key for the 37 established species distributed within China and one new species described herein in *Dendrocalamus*.

### ﻿Key to the species of *Dendrocalamus* in China

**Table:** 

1	Culms apically nodding, not pendulous; branching from lower nodes. Three dominant branches. Foliage leaves small; auricles small. Pseudo-spikelets 1–80, clustered on each node of flowering branches into a spicate globose mass. Florets 1–5; lemma mucronate or long mucronate; lodicules absent, occasionally 1 or 2; stigmas 1–3 (I. subg. Dendrocalamus)	**2**
–	Culms apically pendulous, basally without branches, dominant branches one or indistinct. Foliage leaves usually large; auricles small to vestigial. Pseudo-spikelets 1–30 on nodes of flowering branches. Florets 2–8; lemma not mucronate; lodicules absent to 1; stigmas 1 (II. subg. Sinocalamus)	**18**
2	Culm leaf auricles present	**3**
–	Culm leaf auricles absent or inconspicuous	**6**
3	Culm leaf auricles with bristle-like oral setae (length ≥ 1 cm)	**4**
–	Culm leaf auricles without bristle-like oral setae (length < 1 cm)	**5**
4	Culm leaf auricle oral setae 1 cm long; foliage leaves 10–15 cm long	***D. barbatus***
–	Culm leaf auricle oral setae 1–2.5 cm long; foliage leaves 15–30 cm long	***D. sikkimensis***
5	Culm leaf auricles narrow-oblong, undulate, oral setae 6 mm long; culm height 15–20 m	***D. asper***
–	Culm leaf auricles 5 mm long, 1 mm wide, oral setae easily deciduous; culm height 8–15 m	***D. membranaceus***
6	Culm blade reflexed	**7**
–	Culm blade erect or nearly erect	**10**
7	Culm wall thickness 1.2–2 cm. Culm leaf auricles absent, ligule 1 mm high. Pseudo-spikelet 10–15, clusters diameter 1–2 cm. Style 4.5 mm long, stigma 1	***D. hamiltonii***
–	Culm wall thickness ca. 3 cm. Culm leaf auricles present, but small, ligule 6–10 mm high. Pseudo-spikelet 5–25, clusters diameter 1.3–1.8 cm. Style 3 mm long, stigma 1 or 2	***D. brandisii***
8	Branching starting from 6^th^–7^th^ node, culm leaf auricles undulate-shrunken	***D. liboensis***
–	Branching starting from 7^th^–11^th^ node, culm leaf auricles absent	***D. tsiangii***
9	Culm leaf ligule 1–3 mm high, three dominant branches. Pseudo-spikelet clusters diameter 2.5–5 cm, florets 2–4; glumes 2 or more	***D. strictus***
–	Culm leaf ligule ca. 10 mm high. One dominant branch. Pseudo-spikelet clusters diameter 1–3.5 cm, florets 4; glumes 1	***D. menghanensis***
10	Pseudo-spikelets forming globose clusters (diameter 2.5–5 cm)	**11**
–	Pseudo-spikelets not forming globose clusters (diameter 0.6–3.5 cm)	**12**
11	Culm base nodes 3–7 with aerial roots, culm blade erect, foliage leaf sheath glabrous	***D. tibeticus***
–	Culm base without aerial roots, culm blade erect or reflexed, foliage leaf sheath pubescent	**13**
12	Culm blade erect, branching starting 0.2–0.5 m above ground. Pseudo-spikelet clusters diameter 0.6–1.5 cm, glumes 1	***D. atroviridis***
–	Culm blade erect to spreading, branching starting about 2 m above ground. Pseudo-spikelet clusters diameter 1.5–3.5 cm, glumes 2	***D. jinghongensis***
13	Culm sheaths deciduous, sparsely brown hairy; culm leaf ligule 2–4 mm high, margin denticulate. Branching starting from 8^th^–9^th^ node, branches several and usually equal	***D. yingjiangensis***
–	Culm sheath persistent, densely black bristles; culm leaf ligule ca. 1 mm high, margin entire. Branching from base, branches several with one dominant branch	***D. puerensis***
14	Culm leaf auricles absent. Pseudo-spikelet 5–15 on nodes of flowering branches, clusters diameter 1.5–3.0 cm, florets 4	***D. xishuangbannaensis***
–	Culm leaf auricles present, 1–3 mm high. Pseudo-spikelet 5–25 on nodes of flowering branches, clusters diameter 1–1.8 cm, florets 2 or 3	***D. birmanicus***
15	Culm sheath deciduous; culm leaf ligule ca. 2 mm high. Pseudo-spikelet comprising 3 or 4 florets	***D. bambusoides***
–	Culm sheath persistent; culm leaf ligule ca. 1 cm high. Pseudo-spikelet comprising 4–5 florets	***D. menglongensis***
16	Culm blade base slightly constricted inward; oral setae 7–10 mm long. Foliage blade glabrous on both surfaces	***D. farinosus***
–	Culm blade base outwardly extended; oral setae 1–2 mm long. Foliage blade glabrous adaxially, white-pubescent abaxially	***D. pulverulentus***
17	Pseudo-spikelets 20–35 on nodes of flowering branches, clusters diameter 2.5–3.2 cm. Florets 2–3, style 1	***D. parishii***
–	Pseudo-spikelets 30–40 on nodes of flowering branches, clusters diameter 1–3.2 cm. Florets 4 or 5, style 1–2	***D. semiscandens***
18	Basal nodes (at least 1–5) ringed with aerial roots or with aerial root traces	**19**
–	Basal nodes without aerial roots or aerial roots inconspicuous	**21**
19	Culm leaf auricles present (small, linear, with oral setae); culm leaf ligule 15 mm high, margin deeply lobed; pseudo-spikelets 8–12 mm long	***D. wazibii***
–	Culm leaf auricles absent; culm leaf ligule ≤ 15 mm high; pseudo-spikelets 1–3.5 cm long	**20**
20	Young internodes at first densely covered with brown felt-like tomentum, later turning silvery-white; basal nodes 1–8 ringed with aerial roots; culm leaf ligule 5–7 mm high	***D. tomentosus***
–	Internodes with appressed small white hairs; basal nodes 4–5 ringed with aerial roots; culm leaf ligule 6–10 mm high; leaf blades up to 10 cm wide	***D. peculiaris***
21	Culm leaf auricles present (not extremely small or absent, with recognisable form and oral setae)	**22**
–	Culm leaf auricles absent or very small (length ≤ 3 mm, deciduous, oral setae absent)	**26**
22	Culm leaf auricles narrow-oblong, oral setae 0.6–0.8 cm long. Young culms densely covered with a white powdery coating, glabrous	***D. longiauritus***
–	Culm leaf auricles small and appressed, oral setae ca. 10 mm long. Young culms densely covered with prickly hairs	***D. sanjiangensis***
23	Culm leaf auricles connected to base of culm blade, somewhat reflexed; culm diameter 20–30 cm, height 20–30 m. Anthers 6.5 mm long, pistil ca. 1 cm long	***D. giganteus***
–	Culm leaf auricles small (length 0.5–2 cm, width 1 mm); culm diameter 10–12 cm, height 17–18 m. Anthers 4–6 mm long, pistil 1–1.5 cm long	***D. jianshuiensis***
24	Culm leaf auricles narrowly elliptic (ca. 2 mm long, 0.5 mm wide); culm height 8–10 m, diameter ca. 8 cm; leaf blades 14–45 cm long	***D. pulverulentoides***
–	Culm leaf auricles small (5 mm long, 1 mm wide); culm height 20–25 m, diameter 15–30 cm, leaf blades 15–50 cm long	***D. latiflorus***
25	Culm sheath deciduous, culm leaf auricles 5 mm tall. Pseudo-spikelets 1–1.6 cm long, florets 5–7	***D. yunnanicus***
–	Culm sheath persistent, culm leaf auricles inconspicuous. Pseudo-spikelets 3–3.5 cm long, florets 5	***D. sinicus***
26	Culm blade reflexed, culm leaf ligule absent. Pseudo-spikelets 1.7–2.4 cm long, 5–8 florets, culm height 10–12 m, diameter 10 cm	***D. pachystachys***
–	Culm blade erect, culm leaf ligule 3 mm high. Pseudo-spikelets 1–1.3 cm long, 4 florets, culm height 20 m, diameter 10–15 cm	***D. fugongensis***
27	Culm blade erect, auricles absent, ligule 1–2 mm high. Foliage blades 10–25 cm long, 1.5–3 cm wide. Pseudo-spikelets 2–5 on nodes of flowering branches	***D. calostachyus***
–	Culm blade reflexed, auricles 3 mm tall, easily deciduous, ligule 3–8 mm high. Foliage blades 23–30 cm long, 2.5–6.5 cm wide. Pseudo-spikelets 5–10 on nodes of flowering branches	***D. minor***

## Supplementary Material

XML Treatment for Dendrocalamus wazibii

## ﻿Additional information

### Use of AI

No use of AI was reported.

### Author ORCIDs

Jian-Wei Li https://orcid.org/0009-0004-5362-982X

Mao-Sheng Sun https://orcid.org/0009-0003-7379-3988

Hao-Feng Bao https://orcid.org/0009-0009-0129-1384

Chao-Mao Hui https://orcid.org/0009-0004-5015-2085

Wei-Yi Liu https://orcid.org/0000-0001-7115-8030

### Data availability

All of the data that support the findings of this study are available in the main text.

## References

[B1] Bamboo Phylogeny Group (2012) An updated tribal and subtribal classification of the bamboos (Poaceae: Bambusoideae). In: Gielis J, Potters G (Eds) Proceedings of the 9^th^ World Bamboo Congress, 3–27.

[B2] Chen SH Chen RS Huang KF Guo HZ (2013) *Dendrocalamus longiauritus* S.H.Chen, K.F.Huang et R.S.Chen, a new *Dendrocalamus* species of Bambusoideae from China. Plant Science Journal 31(6): 536–539. 10.3724/SP.J.1142.2013.60536

[B3] IUCN (2024) Guidelines for Using the IUCN Red List Categories and Criteria, Ver. 16. Prepared by the Standards and Petitions Committee. https://www.iucnredlist.org/documents/RedListGuidelines.pdf [Accessed on 8 May 2025]

[B4] Keng PC Wang ZP (1996) Flora Reipublicae Popularis Sinicae (Vol. 9, Vol. 1). Science Press, Beijing, 152–193.

[B5] Li DZ Hsueh C (1988) A study on the genus *Dendrocalamus* Nees from China (I). Journal of Bamboo Research 7(3): 1–19.

[B6] Li DZ Wang ZP Zhu ZD Xia NH Jia LZ Guo ZH Yang GY Stapleton CMA (2006) *Dendrocalamus* (Poaceae). In: Wu ZY Raven PH Hong DY (Eds) Flora of China, Vol.22. Science Press, Beijing; Missouri Botanical Garden Press, St. Louis, 39–46.

[B7] Mao W Yang HQ Li DZ (2009) *Dendrocalamus xishuangbannaensis* (Poaceae: Bambusoideae), a new species from Yunnan, China. Annales Botanici Fennici 46: 574–576. 10.5735/085.046.0612

[B8] Nguyen VT Le VL (2012) *Dendrocalamus nianhei* (Poaceae: Bambusoideae), a new species from northern Vietnam. Annales Botanici Fennici 49: 428–431. 10.5735/085.049.0621

[B9] Nguyen VT Xia NH (2013) A new bamboo species of *Dendrocalamus* Nees (Poaceae: Bambusoideae) from Yen Bai and Phu Tho provinces, Vietnam. Adansonia 35: 55–60. 10.5252/a2013n1a5

[B10] Nguyen VT Xia NH Le VL (2010) *Dendrocalamus parvigemma* sp. nov. (Gramineae: Bambusoideae) from Vietnam. Nordic Journal of Botany 29: 221–223. 10.1111/j.1756-1051.2010.01002.x

[B11] Nguyen VT Le VL Vu VD Xia NH (2012) *Dendrocalamus velutinus* N.H. Xia, V.T. Nguyen & V.D. Vu (Poaceae), a new species from Vietnam. Candollea 67: 255–259. 10.15553/c2012v672a6

[B12] Nguyen VT Xia NH Nguyen HN Le VL (2013a) Three large-stature bamboo species of *Dendrocalamus* (Poaceae: Bambusoideae) from northern Vietnam. Blumea 57: 253–262. 10.3767/000651913X664595

[B13] Nguyen VT Le VL Vu VD Xia NH (2013b) *Dendrocalamus longiligulatus* sp.nov. (Poaceae: Bambusoideae) from Son La, Dien Bien province, Vietnam. Nordic Journal of Botany 31: 607–611. 10.3767/000651913X664595

[B14] Nguyen VT Xia NH Le VL (2014) *Dendrocalamus longivaginatus* (Poaceae, Bambusoideae), A new species from Vietnam. Novon 23: 302–306. 10.3417/2012001

[B15] Nguyen HN Nguyen VT Nguyen AD Vien N Tien PQ Tran VT (2017a) *Dendrocalamus dienbienensis* (Poaceae: Bambusoideae), a new species from Northern Vietnam. Phytotaxa 327(3): 290–296. 10.11646/phytotaxa.327.3.9

[B16] Nguyen HN Nguyen VT Le VL Tran VT Vien N (2017b) *Dendrocalamus phuthoensis* (Poaceae: Bambusoideae), a new species from Phu Tho province, Vietnam. Phytotaxa 296(3): 274–280. 10.11646/phytotaxa.296.3.6

[B17] Shi M Wang SG He CH Zhang XB Yang YM (2024) *Dendrocalamus puerensis*, a New Species of *Dendrocalamus* from South Yunnan, China. Journal of West China Forestry Science 53(4): 72–75.

[B18] Wang PY Li DZ (2019) *Dendrocalamus menghanensis* (Poaceae, Bambusoideae), a new woody bamboo from Yunnan, China. PhytoKeys 130: 143–150. 10.3897/phytokeys.130.33948 31534402 PMC6728398

[B19] Wang PY Zhang YX Li DZ Liu JP (2016) *Dendrocalamus jinghongensis* (Poaceae, Bambusoideae), another new woody bamboo from Yunnan, China. Phytotaxa 272(3): 209–214. 10.11646/phytotaxa.272.3.5

[B20] Yang HQ Xie N Cui YZ Li DZ (2015) *Dendrocalamus yingjiangensis* (Poaceae), a new species of bamboo from western Yunnan Province of China. Annales Botanici Fennici 52: 262–264. 10.5735/085.052.0319

[B21] Yang HQ Xie N Sun MS Xu T Li DZ (2016) *Dendrocalamus atroviridis* (Poaceae: Bambusoideae, Bambuseae), a new species from Southwest China. Phytotaxa 243(2): 170–174. 10.11646/phytotaxa.243.2.7

[B22] Yi TP Shi JY Ma LS Wang HT Yang L (2008) Iconographia Bambusoidearum Sinicarum. Science Press, Beijing, 415–505.

